# Human Concentrative Nucleoside Transporter 3 Transfection with Ultrasound and Microbubbles in Nucleoside Transport Deficient HEK293 Cells Greatly Increases Gemcitabine Uptake

**DOI:** 10.1371/journal.pone.0056423

**Published:** 2013-02-18

**Authors:** Robert J. Paproski, Sylvia Y. M. Yao, Nicole Favis, David Evans, James D. Young, Carol E. Cass, Roger J. Zemp

**Affiliations:** 1 Department of Electrical and Computer Engineering, University of Alberta, Edmonton, Alberta, Canada; 2 Department of Physiology, University of Alberta, Edmonton, Alberta, Canada; 3 Department of Medical Microbiology and Immunology, University of Alberta, Edmonton, Alberta, Canada; 4 Department of Oncology, Cross Cancer Institute, University of Alberta, Edmonton, Alberta, Canada; 5 Membrane Protein Disease Research Group, University of Alberta, Edmonton, Alberta, Canada; Kyushu University, Japan

## Abstract

Gemcitabine is a hydrophilic clinical anticancer drug that requires nucleoside transporters to cross plasma membranes and enter cells. Pancreatic adenocarcinomas with low levels of nucleoside transporters are generally resistant to gemcitabine and are currently a clinical problem. We tested whether transfection of human concentrative nucleoside transporter 3 (hCNT3) using ultrasound and lipid stabilized microbubbles could increase gemcitabine uptake and sensitivity in HEK293 cells made nucleoside transport deficient by pharmacologic treatment with dilazep. To our knowledge, no published data exists regarding the utility of using hCNT3 as a therapeutic gene to reverse gemcitabine resistance. Our ultrasound transfection system - capable of transfection of cell cultures, mouse muscle and xenograft CEM/araC tumors - increased hCNT3 mRNA and ^3^H-gemcitabine uptake by >2,000– and 3,400–fold, respectively, in dilazep-treated HEK293 cells. Interestingly, HEK293 cells with both functional human equilibrative nucleoside transporters and hCNT3 displayed 5% of ^3^H-gemcitabine uptake observed in cells with only functional hCNT3, suggesting that equilibrative nucleoside transporters caused significant efflux of ^3^H-gemcitabine. Efflux assays confirmed that dilazep could inhibit the majority of ^3^H-gemcitabine efflux from HEK293 cells, suggesting that hENTs were responsible for the majority of efflux from the tested cells. Oocyte uptake transport assays were also performed and provided support for our hypothesis. Gemcitabine uptake and efflux assays were also performed on pancreatic cancer AsPC-1 and MIA PaCa-2 cells with similar results to that of HEK293 cells. Using the MTS proliferation assay, dilazep-treated HEK293 cells demonstrated 13-fold greater resistance to gemcitabine compared to dilazep-untreated HEK293 cells and this resistance could be reversed by transfection of hCNT3 cDNA. We propose that transfection of hCNT3 cDNA using ultrasound and microbubbles may be a method to reverse gemcitabine resistance in pancreatic tumors that have little nucleoside transport activity which are resistant to almost all current anticancer therapies.

## Introduction

Gemcitabine is a nucleoside analog currently used to treat various solid tumors [1]. Gemcitabine is a hydrophilic molecule and requires specialized membrane proteins called nucleoside transporters (NTs) to efficiently cross plasma membranes [2]. Human equilibrative nucleoside transporters (hENT1/2/3/4) are bidirectional NTs that transport nucleosides according to their concentration gradients [3]. Human concentrative nucleoside transporters (hCNT1/2/3) are symporters that co-transport nucleosides and Na^+^ (and/or H^+^ for hCNT3) inside cells [4]. hENT1/2 and hCNT1/3 are the plasma membrane NTs most efficient at gemcitabine transport [5].

Most clinical studies analyzing the relationship between gemcitabine sensitivity and nucleoside transporter levels in tumors have focused on hENT1 [6]–[10]. Pancreatic tumor levels of hENT1 (detected by immunohistochemistry) vary considerably with 22%, 37%, and 40% of tumors having no, high, and low detectable hENT1 staining, respectively [6]. Two separate prospective clinical studies have both demonstrated that pancreatic cancer patients with low tumor hENT1 staining have significantly reduced disease-free survival and overall survival (approximately 2- to 3- fold) compared to patients with high hENT1 tumor staining [6], [8]. Pancreatic tumor hENT1 mRNA levels have also correlated with disease-free survival and overall survival, demonstrating the importance of hENT1 for gemcitabine sensitivity [7], [9]. Unfortunately, few therapeutic options are currently available for pancreatic cancer patients with low hENT1 levels since gemcitabine is one of the very few drugs capable of increasing survival times for these patients.

In theory, gene therapy could be used to help treat pancreatic cancers with low nucleoside transporter activity. Previous studies have demonstrated that transfection of DNA encoding hENT1 or hCNT1 in cultured cancer cells with low NT activity can significantly decrease gemcitabine resistance [11], [12]. hCNT3 is a better candidate as a therapeutic gene to overcome gemcitabine resistance, since when expressed in *Xenopus laevis* oocytes, hCNT3 demonstrated 4.4- to 219-fold greater levels of gemcitabine uptake than any other hNT [5]. However, attempts to introduce hCNT3 into a genetically transport-deficient cell line using established transfection methods that were successful with hCNT1 and hCNT2 [13], [14] proved unsuccessful (unpublished results).

In the current study, ultrasound and lipid-stabilized microbubbles (LSM) were used to transfect DNA into cells through a process known as sonoporation [15]. Sonoporation involves ultrasound exposure of cells which may induce pore formation in membranes, allowing free passage of drugs/nucleic acids into or out of sonicated cells. Low MHz ultrasound fields can be broad or focused and can penetrate multiple centimeter depths in tissues, allowing non-invasive and region specific transfection in relatively deep tissues. Previous studies involving sonoporation have demonstrated *in vitro* and *in vivo* transfection efficiencies as high as 95% and 67%, respectively [16], [17]. The majority of studies using ultrasound and microbubbles to transfect cells analyze the transfection efficiency of various sonoporation protocols using standard reporter genes (fluorescent proteins, luciferase, *etc*). The current study assesses the utility of sonoporation for gene therapy of hCNT3 to overcome gemcitabine resistance and the advantages and challenges of this technique will be discussed.

## Materials and Methods

### Materials

Unless otherwise stated, all chemicals were purchased from Sigma-Aldrich (St. Louis, MO, USA). Phosphate buffered saline (PBS), fetal bovine serum (FBS), Dulbecco’s modified Eagle’s medium (DMEM), OptiMEM, and trypsin were purchased from Gibco (Carlsbad, CA, USA). pIRES2-EGFP plasmid was obtained from Clontech Laboratories, Inc. (Mountain View, CA, USA). Perfluoropropane gas was purchased from Electronic Fluorocarbons, LLC (Hopkinton, MA, USA). HEK293 cells (derived from human embryonic kidney) were obtained from American Type Culture Collection (Manassas, VA, USA). CEM/araC8 cells (a drug-resistant mutant from CCFR-CEM cells, derived from a human lymphoblastic leukemia) were obtained from Dr. B. Ullman and their absence of nucleoside transport activity and resistance to gemcitabine has been described previously [2], [13]. Tritiated and unlabeled forms of gemcitabine were obtained from Moravek Biochemicals (Brea, CA, USA) and Eli Lilly (Indianapolis, Indiana, USA), respectively. D-Luciferin was obtained from GoldBiotechnology, Inc. (St. Louis, MO, USA). Lipofectamine 2000 was purchased from Invitrogen (Carlsbad, CA, USA).

### Preparation of Lipid-stabilized Microbubbles (LSM)

LSM were prepared as previously described [16]. Briefly, a lipid stock of 1,2-dipalmitoyl-*sn*-glycero-3-phosphocholine (27 mg), 1,2-dipalmitoyl-*sn*-glycero-3-phosphoethanolamine (3 mg), and glucose (1 g) was dissolved in 10 mL PBS, heated at ∼90°C for 30 minutes, and stored at 4°C. For each batch of LSM, 350 µL lipids stock was added to a 1.5-mL centrifuge tube and mixed with 5 µL bovine serum albumin (10% w/v) and 50 µL glycerol. The headspace of the tube was filled with perfluoropropane gas and LSM were created by shaking the tube with a dental amalgamator (D650 Amalgamator) for 30 seconds.

### Ultrasound and Microbubble Transfection of Cultured HEK293 Cells

**Figure 1:**
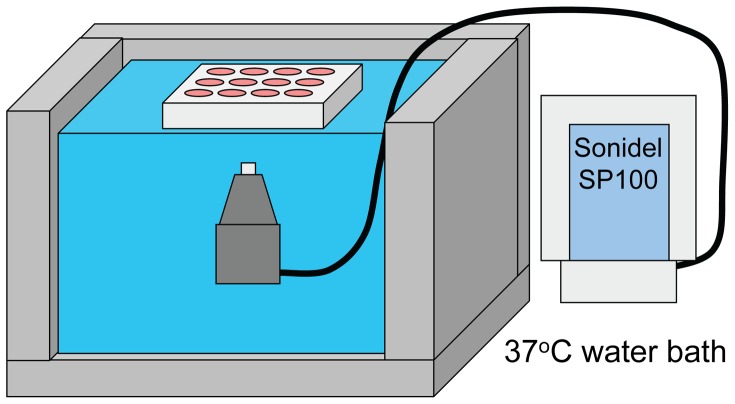
Ultrasound/LSM transfection setup. The bottom of the 12-well plate with cells was submerged in a 37°C water bath. The SP100 ultrasound transducer was placed underneath the plate and ultrasound was emitted upwards into the bottom of each well.

HEK293 cells were inoculated in 12-well plates at 4×10^5^ cells/well and cultured for three days in DMEM with 10% (v/v) FBS. Fully confluent HEK293 cells were then washed twice with OptiMEM and incubated with 0.4 mL OptiMEM/well with or without 12 µg/mL pIRES2-EGFP-hCNT3 DNA for 30 minutes at 37°C. LSM (4×10^7^ LSM/mL) were added and mixed in the medium of each well. Plates were individually placed into our 37°C water bath transfection apparatus (Fig. 1) and ultrasound (1 MHz, 100 Hz pulse repetition frequency, 0.5 W/cm^2^, 25% duty cycle) from a SP100 system (Sonidel, Ireland) was applied to the bottom of desired wells for 1 minute. The ultrasound transducer was slowly moved in a circular motion by hand to ensure all cells in wells received ultrasound. Plates were then placed back in a 37°C incubator for 1 hour followed by addition of 1 mL/well DMEM with 10% FBS. Cells were placed back in the 37°C incubator and used for experiments the following day.

Transfection efficiency was analyzed the following day by fluorescence microscopy. At least six images of transfected cells were taken from different wells and images were analyzed with Metamorph software (Molecular Devices, LLC, Sunnyvale CA, USA). Image area of total cells and cells expressing green fluorescent protein were determined using region of interest analysis tools and transfection efficiency was calculated as 100× transfected cell area/total cell area.

### Ultrasound and Microbubble Transfection of Muscle and Tumor

All animal procedures were approved by the University of Alberta’s biosciences animal services animal care and use committee (permit number: 700/06/12). For each procedure, mice were under isoflurane anesthesia to minimize animal discomfort. Severe combined immunodeficiency hairless outbred (SHO) mice (5–7 week old) were obtained from Charles River (Wilmington, MA, USA). For transfection of mouse muscle tissue, both back legs of an isoflurane-anesthetized mouse were injected with 50 µL LSM mixed with 50 µg pSJ7-luciferase DNA. Only the back right leg of the mouse was exposed to ultrasound from the SP100 system (1 MHz, 100 Hz pulse repetition frequency, 1.9 W/cm^2^, 25% duty cycle, 3 minutes) using ultrasound contact gel for acoustic coupling between the transducer and mouse. The mouse recovered from anesthesia and was imaged for bioluminescence three days later.

For transfection of tumors, SCID hairless outbred (SHO) mice were first injected subcutaneously in the back flank with 3×10^5^ CEM/araC8 cells in 0.1 mL growth medium with 25% (v/v) matrigel (BD Biosciences, Franklin Lakes, NJ, USA). When the tumors were at least 7 mm in diameter, mice were anesthetized with isoflurane and injected intratumorally with either 50 µL PBS with 50 µg pSJ7-luciferase DNA with or without LSM. Tumors were sonicated with the SP100 system (1 MHz, 100 Hz pulse repetition frequency, 1.9 W/cm^2^, 25% duty cycle, 3 minutes). Ultrasound coupling was achieved using a small water filled plastic container with a thin plastic film bottom which was in contact with the tumor with ultrasound coupling gel in between the tumor and plastic film. Mice recovered from anesthesia and were imaged for bioluminescence three to seven days later.

Bioluminescent imaging was performed using the IVIS® Spectrum Optical Imaging system (Caliper Life Sciences, Hopkinton, MA, USA). Prior to imaging, isoflurane-anesthetized mice were given an intraperitoneal injection of a 0.2 mL luciferin solution. The filter-sterilized luciferin solution was made to a concentration of 15 mg/mL using D-luciferin potassium salt dissolved in PBS. Mice were then placed onto the IVIS® Spectrum imager stage and were imaged for bioluminescence every minute for up to 20 minutes with 1-second exposure durations using the same settings for each imaging procedure.

### Nucleolside Transporter mRNA Quantification with TaqMan® Real-time PCR

HEK293 cells exposed to ultrasound and LSM with or without pIRES2-EGFP-hCNT3 were harvested by trypsinization the day following transfection (see above for transfection protocol). AsPC-1 and MIA PaCa-2 cells were inoculated in 24-well plates at 5×10^4^ cells/well and three days later some cells were transfected with pIRES2-EGFP-hCNT3 using lipofectamine 2000 according to manufacturers’ instructions. Cells were lysed by multiple passes through a syringe with a 27-gauge needle and mRNA was purified using a RNeasy mini kit (Qiagen, Venlo, Netherlands; see manufacturer’s instructions for purification procedure). Any remaining pIRES2-EGFP-hCNT3 plasmids in 30 µL RNA samples were digested with 0.5 µg DNase I for 10 minutes at room temperature and were purified again using the “RNA cleanup” procedure with the RNeasy mini kit. The reverse transcription and TaqMan procedures have been described previously [18]. Briefly, RNA (2 µg) was reverse transcribed into cDNA using a TaqMan® reverse transcription kit (Applied Biosystems, Foster City, CA, USA) according to manufacturer’s instructions. cDNA (2 µL) was mixed with 2× TaqMan Universal Master mix II (Applied Biosystems, Foster City, CA, USA), probes and primers for hENT1, hENT2, hCNT1, hCNT3, or glyceraldehyde-3-phosphate dehydrogenase (GAPDH; see [18] for description of probes and primers), and water (to dilute master mix to 1× concentration) and 20 µL of solution was added to each well of 96-well plates. Plates were analyzed by an Applied Biosystems 7900 HT Fast Real Time PCR system (Applied Biosystems, Foster City, CA, USA). GAPDH was determined to correct for cDNA loading and, for each cell line, nucleoside transporter mRNA levels were normalized to that of hCNT3 in untransfected cells using the ΔΔC_T_ method [19].

### ^3^H-Gemcitabine Uptake in Cells Transfected with or without hCNT3

HEK293 cells in 12-well plates were exposed to ultrasound and LSM with or without pIRES2-EGFP-hCNT3 (see above for transfection protocol). AsPC-1 and MIA PaCa-2 cells were inoculated in 12-well plates at 1×10^5^ cells/well. Three days later, cells were transfected with pIRES2-EGFP-hCNT3 using lipofectamine 2000 according to manufacturers’ instructions. The following day, cells were washed once with PBS (1 mL/well) and then incubated with 0.5 mL/well transport buffer (20 mM Tris, 3 mM K_2_HPO_4_, 5 mM glucose, 145 mM NaCl, 1 mM MgCl_2_, and 1.2 mM CaCl_2_) containing 50 nM ^3^H-gemcitabine with or without 100 µM dilazep which inhibits endogenous hENT activity. Cells were incubated at 37°C for one hour followed by three washes with PBS (1 mL/well). Cells were lysed by incubation with 0.5 mL/well 0.5 M KOH for one hour. Protein levels in cell lysates were analyzed using the Bio-Rad Protein Assay (Bio-Rad Laboratories, Hercules, CA).

^3^H-Gemcitabine uptake values (pmol/mg protein/hour) in each well are summations of ^3^H-gemcitabine uptake from hCNT3-transfected and untransfected cells (i.e., ^3^H-gemcitabine uptake/well = [(fraction of cells which are transfected)*(^3^H-gemcitabine uptake in transfected cells)]+[(fraction of cells which are untransfected)*(^3^H-gemcitabine uptake in untransfected cells)]. Since both the transfection efficiency (see above) and ^3^H-gemcitabine uptake values in untransfected cells are known, we can determine ^3^H-gemcitabine uptake in hCNT3-transfected cells by simple algebra. Presented ^3^H-gemcitabine uptake values have been corrected for transfection efficiency using the equation above.

### ^3^H-Gemcitabine Efflux from Cells Incubated with or without Dilazep

HEK293, AsPC-1, and MIA PaCa-2 cells were inoculated in 12-well plates at 4×10^5^, 1×10^5^, and 1×10^5^ cells/well, respectively. Four days later, cells were washed once with PBS (1 mL/well) and then incubated with 0.5 mL/well transport buffer (see above) containing 50 nM ^3^H-gemcitabine for one hour at 37°C. Cells were then washed twice with PBS with or without 100 µM dilazep (1 mL/well) and then incubated with 1 mL/well PBS with or without 100 µM dilazep. PBS aliquots of 100 µL were taken 5, 15, 30, and 60 min after incubation and analyzed for ^3^H-gemcitabine using a liquid scintillation counter.

### Gemcitabine Toxicity in HEK293 Cells Transfected with or without hCNT3

HEK293 cells were inoculated in 6-well plates at 5×10^5^ cells/well and were transfected with either pIRES2-EGFP or pIRES2-EGFP-hCNT3 using lipofectamine 2000 three days later using the protocol provided by the manufacturer. The following day, transfected HEK293 cells were trypsinized from the 6-well plates and inoculated in 96-well plates at 5×10^4^ cells/well. Each well contained growth medium with or without 1) various concentrations of gemcitabine (10 pM –10 µM), and 2) 10 µM dilazep to inhibit endogenous hENT activity. Cell proliferation assays were performed after three days of drug incubation using the CellTiter 96® AQueous Non-Radioactive Cell Proliferation Assay (MTS assay; Promega, Fitchburg, WI, USA) according to manufacturer’s instructions. Plates with 20 µL/well tetrazolium reagent added were incubated at 37°C for up to 2 hours and were analyzed for 490 nm absorbance using a SpectroMAX 190 plate reader (Molecular Devices, Sunnyvale, CA, USA). Absorbance values from wells without cells (background) were subtracted from those of wells with cells. Data were analyzed with GraphPad Prism 4.0 software using non-linear regression analysis and EC_50_ values were determined as gemcitabine concentrations which provided half of the maximal toxic drug effect.

### ^3^H-Gemcitabine Uptake in Xenopus Laevis Oocytes Injected with hENT1/hCNT3 mRNA

Oocytes are a good model for studying nucleoside transporters because they are naturally deficient of such transporters, but can be made to express them *via* well-controlled micro-injection procedures [20]. ^3^H-Gemcitabine uptake assays with oocytes were performed as previously described but with minor modifications [5]. Briefly, hENT1 and hCNT3 cDNAs were inserted into the oocyte expression vector pGEMHE and RNA from the vector was transcribed using the mMESSAGE mMACHINE® T7 Kit (Ambion). Defolliculated stage VI oocytes were microinjected with 20 nL water with or without 20 ng hENT1/hCNT3 RNA. After four days incubation in Barth’s solution at 18°C, oocytes were incubated for various amounts of time (12 oocytes/time point) with 10 µM ^3^H-gemictabine with or without 1 µM *S*-(4-nitrobenzyl)-6-thioinosine (NBMPR; specific hENT1 inhibitor) in transport buffer containing 100 mM NaCl. Oocytes were then washed six-times with ice cold non-radioactive transport buffer (to remove extracellular radioactivity) and dissolved with 1% (w/v) sodium dodecyl sulfate to determine radioactivity within oocytes by liquid scintillation counting.

## Results

**Figure 2:**
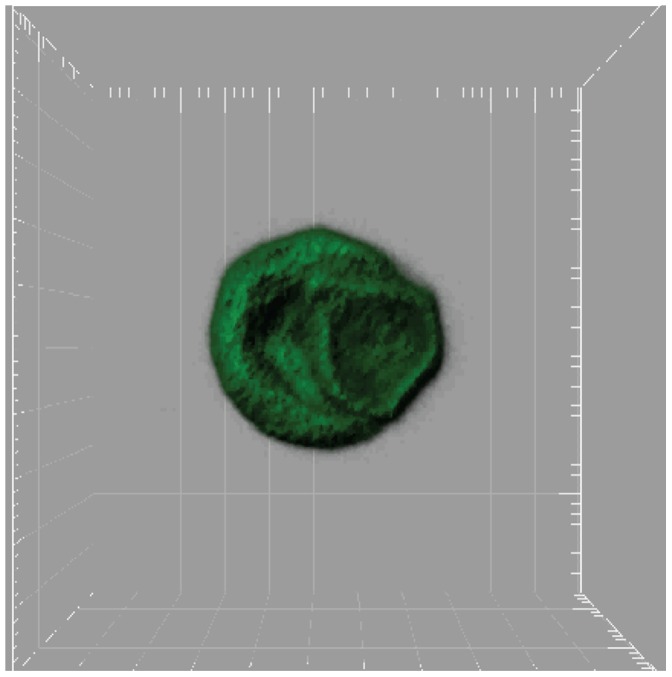
Fluorescence microscopy image of two LSM containing fluorescent water soluble pyranine. Distance between major grid lines is one micrometer. Perfluoropropane gas bubbles in LSM reduces aqueous volume.

LSM were characterized with a Coulter Z2 particle counter and had a mean diameter of 3.6 µm and an average concentration of 4.6×10^9^ LSM/mL. Some LSM were prepared with water soluble pyranine dye and imaged with confocal microscopy (Fig. 2). In general, pyranine levels positively correlated with microbubble size with larger LSM displaying greater levels of fluorescence. Pyranine fluorescence was low for most LSM since the perfluoropropane gas comprised the majority of the volume of the LSM.

**Figure 3:**
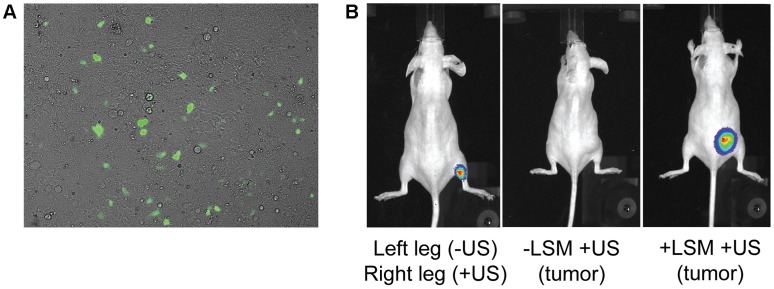
In vitro and in vivo transfection of cells with ultrasound and LSM. (**A**) pIRES2-EGFP-hCNT3 was transfected into cultured HEK293 cells using the setup in Figure 2. (**B**, left) A mouse was injected with LSM and DNA encoding luciferase into both back legs. Ultrasound was only applied to the back right leg and bioluminescence imaging was performed 3 days later. Mice bearing subcutaneous CEM/araC tumors had luciferase encoding DNA with (right) or without (middle) LSM injected into the tumors. Ultrasound was applied to both tumors and bioluminescence imaging was performed 5 days later. Four tumor-bearing mice were imaged with bioluminescence imaging with consistent results. In vivo images suggest efficient transfection only occurs in the presence of both LSM and ultrasound.

For the present study, cultured HEK293 cells were used since they displayed the highest levels of transfection when using the ultrasound setup displayed in Figure 1. Several ultrasound settings were tested for transfecting HEK293 cells and 0.5 W/cm2 with 25% duty cycle for 1 minute provided relatively high transfection rates with low levels of cell detachment. The transfection efficiency for HEK293 cells using the ultrasound settings above and LSM was 2.3±1.3% (Fig. 3A).

Transfection of tissues/tumors with ultrasound and LSM was also assessed using mice. When LSM and DNA encoding luciferase were injected in both back legs of a mouse but with ultrasound only applied to the back right leg, bioluminescence was only observed from the back right leg (Fig. 3B), suggesting that ultrasound was necessary for efficient transfection of muscle tissue. Xenograft CEM/araC tumors in mice only displayed visible bioluminescence if tumors were injected with both DNA encoding luciferase and LSM followed by ultrasound exposure (no bioluminescence was observed without LSM injection), suggesting that LSM were necessary for efficient tumor transfection with ultrasound. Tumor bioluminescence levels were greatest at day 5 (post-ultrasound) and began decreasing at day 7 (data not shown). Both *in vitro* and *in vivo* results suggest various cell types can be transfected with ultrasound and LSM.

**Figure 4:**
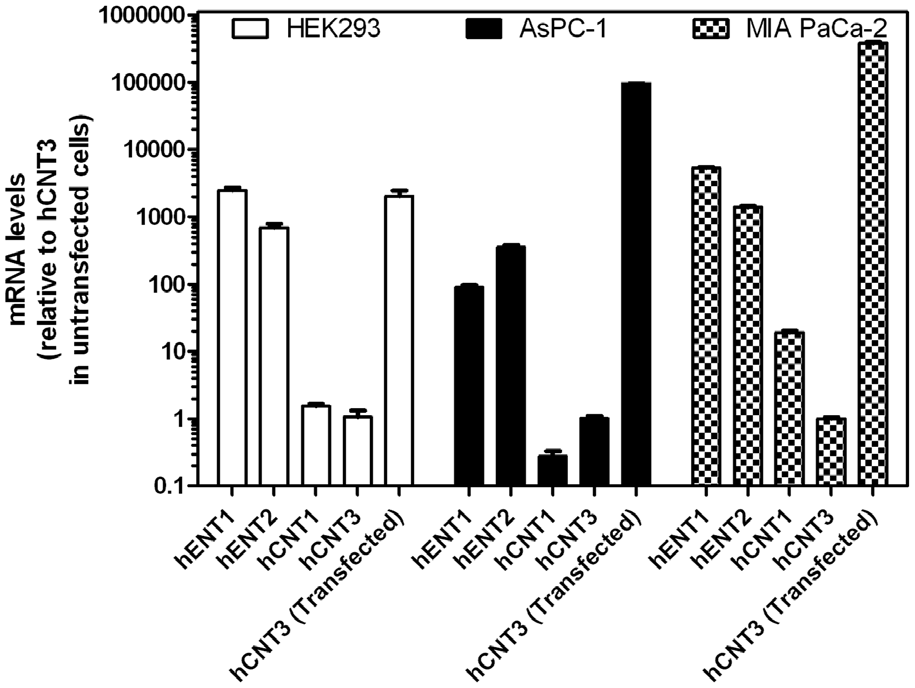
Nucleoside transporter mRNA levels in HEK293, AsPC-1, and MIA PaCa-2 cells with or without transfection of pIRES2-EGFP-hCNT3 DNA. Cultured HEK293 cells were transfected with pIRES2-EGFP-hCNT3 using ultrasound and LSM while AsPC-1 and MIA PaCa-2 cells were transfected using lipofectamine 2000. The following day cells were harvested and analyzed for mRNA levels using real-time PCR with TaqMan® probes and primers. Bars are mean values from three different experiments.

Nucleoside transporter mRNA levels were analyzed in HEK293, AsPC-1, and MIA PaCa-2 cells. For AsPC-1, HEK293, and MIA PaCa-2 cells, respectively, hENT1 and hENT2 mRNA levels were at least 90-, 690-, and 1380-fold larger than mRNA levels of hCNT1 and hCNT3, suggesting that hENT1 and hENT2 were primarily responsible for nucleoside transport activity in these cell lines. hCNT3 mRNA was also analyzed in all three cell lines with or without pIRES2-EGFP-hCNT3 transfection. Transfected HEK293 cells (using ultrasound and LSM), as well as AsPC-1 and MIA PaCa-2 cells (using lipofectamine) displayed 2,000-, 95,000-, and 378,000-fold greater levels of hCNT3 mRNA compared to untransfected cells (Fig. 4), suggesting that hCNT3 levels were greatly increased using the described ultrasound transfection procedures.

**Figure 5:**
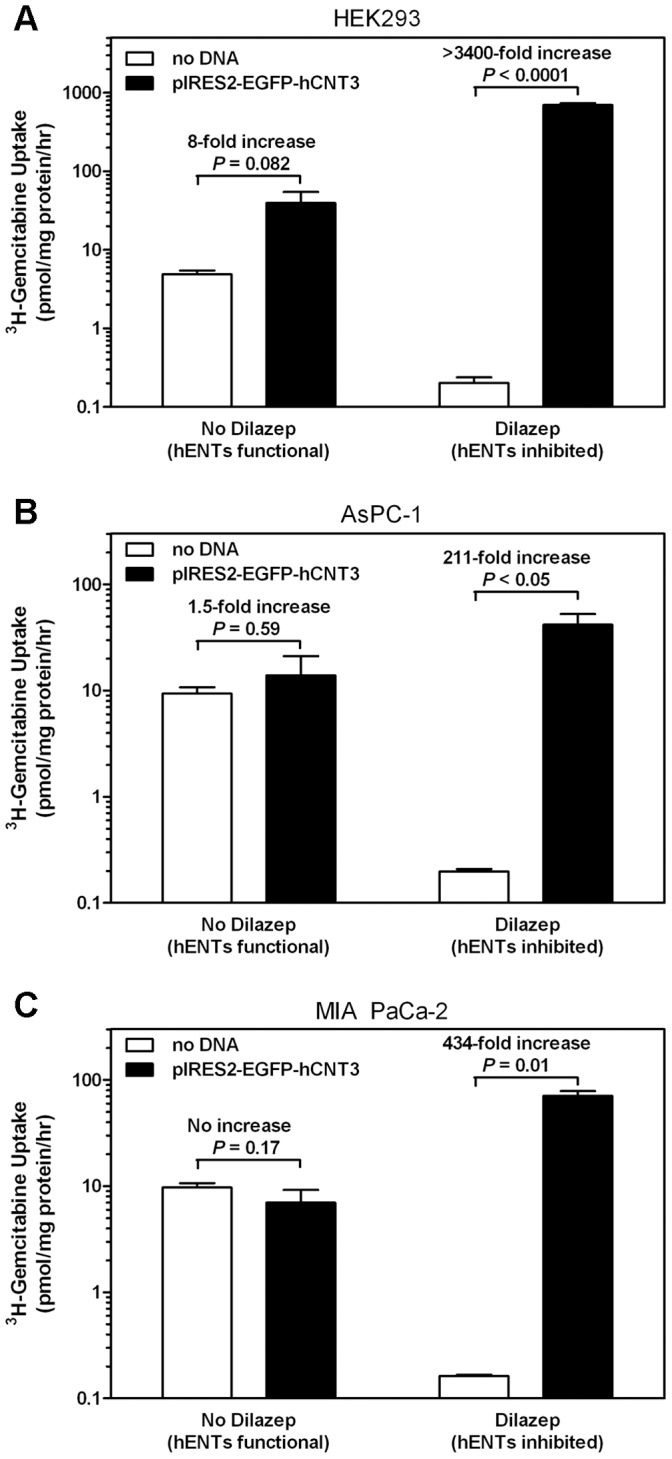
Transfection efficiency corrected ^3^H-gemcitabine uptake in HEK293 (A), AsPC-1 (B), and MIA PaCa-2 (C) cells with or without transfection of hCNT3 cDNA. HEK293 cells in 12-well plates were incubated with buffer containing LSM with or without pIRES2-EGFP-hCNT3 DNA and exposed to ultrasound. AsPC-1 and MIA PaCa-2 cells were transfected with lipofectamine 2000. Uptake assays were performed the following day using 50 nM ^3^H-gemcitabine in the presence or absence of 100 µM dilazep. For all cell lines, hENT inhibition by dilazep significantly decreased ^3^H-gemcitabine uptake whereas cells transfected with hCNT3 cDNA exhibited significantly increased ^3^H-gemcitabine uptake. Uptake values were corrected for transfection efficiency as described in the methods section. Bars represent mean values from three separate experiments (each performed in triplicate).

^3^H-Gemcitabine uptake over 1 hour was compared between HEK293 cells sonicated with LSM with or without pIRES2-EGFP-hCNT3 (Fig. 5A). Untransfected HEK293 cells displayed 24-fold greater ^3^H-gemcitabine uptake in the absence than in the presence of 100 µM dilazep, which blocks hENT1 and hENT2 activities (*P* = 0.01). When correcting for transfection efficiency, ^3^H-gemcitabine uptake in dilazep-untreated HEK293 cells that had been transfected with hCNT3 cDNA increased 8-fold compared to that of untransfected cells. In contrast, ^3^H-gemcitabine uptake increased >3400-fold in dilazep-treated HEK293 cells that had been transfected with hCNT3 cDNA, suggesting that hCNT3 transfection had a 430-fold greater effect, increasing ^3^H-gemcitabine uptake in cells with no functional hENTs compared to cells with functional hENTs. Similar experiments were performed with AsPC-1 and MIA PaCa-2 cells transfected with pIRES2-EGFP-hCNT3 using lipofectamine 2000 (Fig. 5B and 5C). For AsPC-1 and MIA PaCa-2 cells, hCNT3 transfection had, respectively, a 140- and 430-fold greater effect increasing ^3^H-gemcitabine uptake in cells with no functional hENTs compared to cells with functional hENTs.

**Figure 6:**
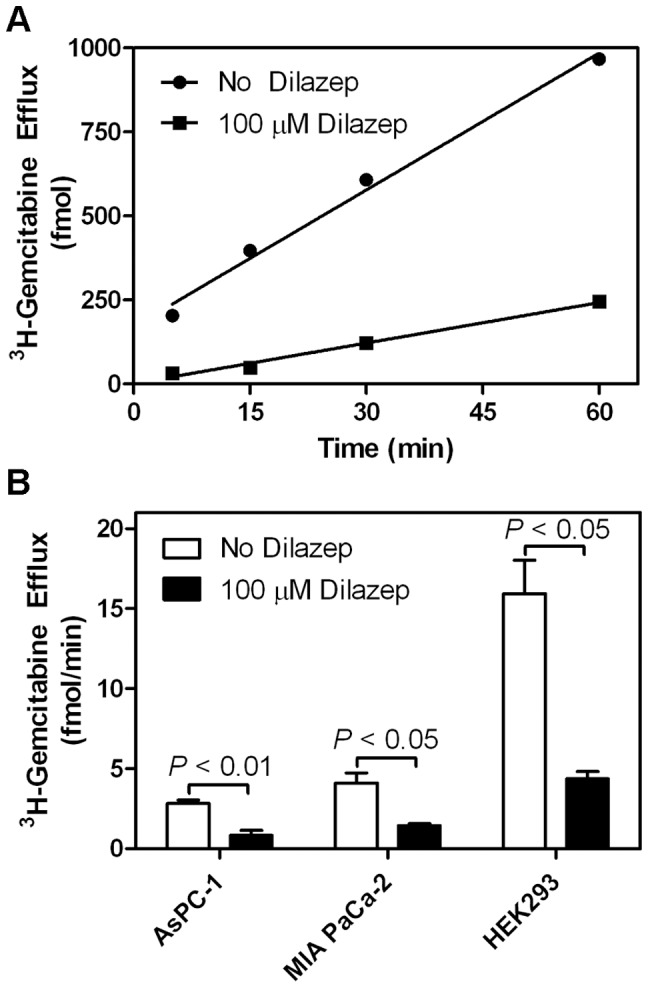
^3^H-Gemcitabine efflux from HEK293, AsPC-1, and MIA PaCa-2 cells. (**A**) HEK293 cells in 12-well plates were incubated with ^3^H-gemcitabine for 60 min and were washed with PBS to remove extracellular ^3^H-gemcitabine. Cells were incubated in PBS with or without 100 µM dilazep and PBS aliquots were taken over 60 min and analyzed for ^3^H-gemcitabine. Shown is a representative experiment. (**B**) ^3^H-Gemcitabine efflux rates for AsPC-1, MIA PaCa-2, and HEK293 cells with or without 100 µM dilazep**.** Bars represent mean values from three different experiments.

Radioactive efflux assays were performed to determine if hENT activity was primarily responsible for ^3^H-gemcitabine efflux from the tested cell lines. Untransfected HEK293, AsPC-1, and MIA PaCa-2 cells were incubated with ^3^H-gemcitabine to load cells with the tracer followed by multiple washing steps to remove extracellular ^3^H-gemcitabine. Cells were incubated in PBS with or without dilazep and effluxed ^3^H-gemcitabine was monitored over 60 min (Fig. 6A). ^3^H-Gemcitabine efflux rates for HEK293, AsPC-1, and MIA PaCa-2 cells incubated in the presence of dilazep were 27, 29, and 35% of those in the absence of dilazep, respectively, suggesting that hENT activity was responsible for the majority of ^3^H-gemcitabine efflux in the tested cell lines (Fig. 6B).

**Figure 7:**
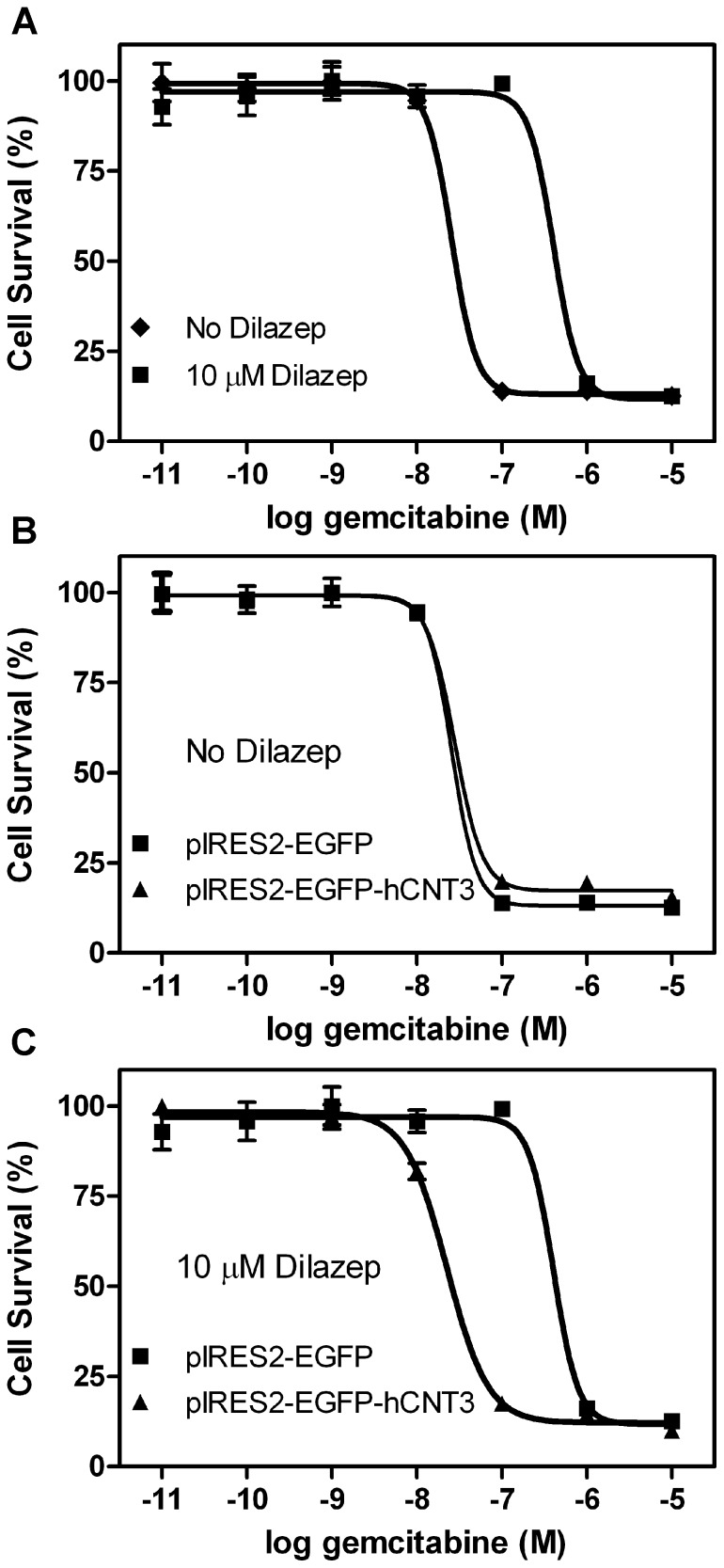
Gemcitabine toxicity in HEK293 cells with or without transfection of hCNT3 cDNA. (**A**) Inhibiting endogenous hENT proteins with 10 µM dilazep significantly increased gemcitabine resistance (340±43 and 26±2 µM EC_50_ values with and without dilazep, respectively). (**B**) Transfection of hCNT3 in HEK293 cells not incubated with dilazep had no affect on gemcitabine sensitivity. (**C**) Cells transfected with hCNT3 cDNA and subsequently incubated with dilazep exhibited significantly increased sensitivity to gemcitabine (35±10 EC_50_ value, *P*<0.005). Representative experiments shown with each experiment performed using 6 replicates. EC_50_ values shown above were determined from averaging the EC_50_ values from three different experiments.

Gemcitabine toxicity over 72 hours was analyzed in the presence or absence of 10 µM dilazep (to block hENT1/2) in HEK293 cells transfected with pIRES2-EGFP (empty vector) or pIRES2-EGFP-hCNT3 using lipofectamine. Cells transfected with pIRES2-EGFP and subsequently treated with dilazep displayed 13-fold greater resistance to gemcitabine compared to dilazep-untreated cells (340±43 and 26±2 nM EC_50_ values, respectively, *P*<0.02, Fig. 7A). There was no significant difference in gemcitabine toxicity in the absence of dilazep between cells transfected with pIRES2-EGFP or pIRES2-EGFP-hCNT3 (26±2 and 29±6 nM EC_50_ values, respectively, Fig. 7B). Treatment of cells that had been transfected with pIRES2-EGFP-hCNT3 with dilazep decreased gemcitabine resistance by 9.8-fold compared to similarily-treated cells that had been transfected with pIRES2-EGFP (35±10 and 340±43 nM EC_50_ values, respectively, *P*<0.02, Fig. 7C).

**Figure 8:**
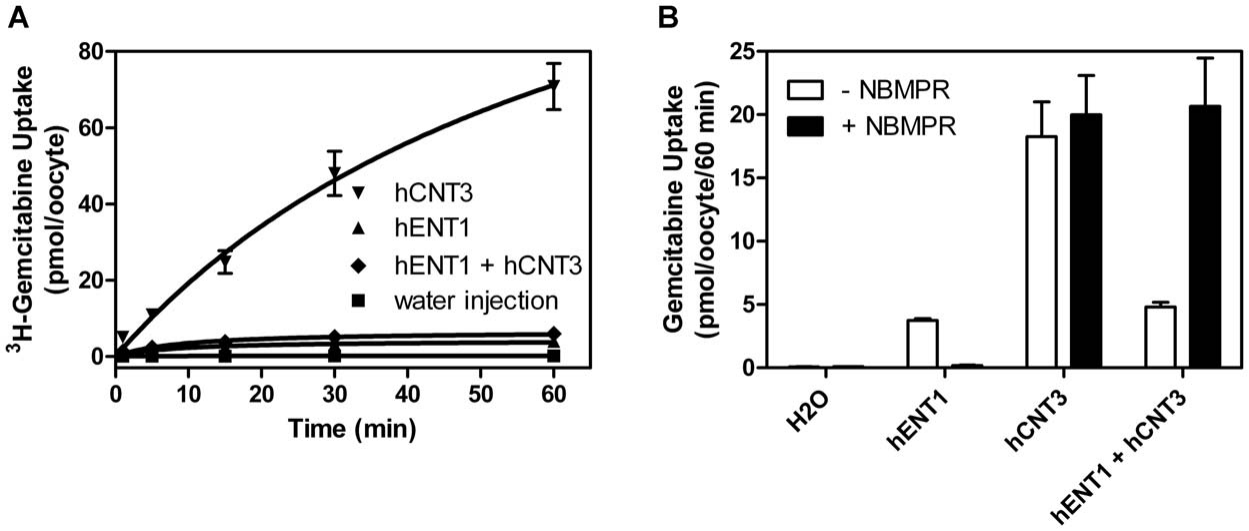
^3^H-gemcitabine uptake in *Xenopus* oocytes with or without production of recombinant hENT1 and hCNT3. (**A**) Time courses of ^3^H-gemcitabine uptake in *Xenopus* oocytes over one hour. Oocytes were microinjected with hENT1/hCNT3 mRNA and uptake assays were performed four days later using 10 µM ^3^H-gemcitabine. (**B**) Oocytes were incubated with or without hENT1 inhibitor NBMPR (1 µM) and ^3^H-gemcitabine uptake was analyzed 60 minutes after ^3^H-gemcitabine incubation. Oocytes producing hCNT3 alone demonstrate the greatest levels of ^3^H-gemcitabine uptake although uptake was greatly reduced upon co-expression of active hENT1. Uptake values for each time point is the mean from 12 oocytes. Error bars not shown when smaller than symbols.

Experiments in which recombinant hCNT3 and hENT1 were produced individually or together in *Xenopus* oocytes confirmed and extended the findings in cultured cells. Recombinant hENT1 was produced in oocytes since, compared to hENT2, hENT1 has higher RNA expression levels in most human tissues and is responsible for the majority of hENT activity in many cancer cells lines [18]. As shown by the time courses of ^3^H -gemcitabine uptake in Fig. 8A, drug accumulation by hCNT3-producing oocytes was much greater than in oocytes producing hENT1 alone, or in oocytes producing both hCNT3 and hENT1. Uptake of ^3^H -gemcitabine in control water-injected oocytes was negligible. The hENT1-specific inhibitor NBMPR had no effect on hCNT3-mediated accumulation of ^3^H -gemcitabine, but markedly reduced uptake in hENT1-producing cells to levels comparable to water-injected cells (Fig. 8B). In oocytes producing both hCNT3 and hENT1, the presence of NBMPR restored ^3^H -gemcitabine accumulation to levels seen in cells producing hCNT3 alone.

## Discussion

With our ultrasound transfection setup, we have achieved an *in vitro* transfection efficiency of 2.3% which is comparable to that of other studies testing the ultrasound pressures used in this study [21]–[23]. Several other studies have demonstrated significantly greater transfection efficiencies (>40%) although greater ultrasound pressures were required which can cause significant levels of cell detachment and cell death [17], [23], [24]. This would have complicated our subsequent ^3^H-gemcitabine uptake assays and thus we used lower ultrasound pressures for transfecting HEK293 cells. However, even with relatively low transfection efficiency, our ultrasound transfection setup was capable of significantly increasing 1) hCNT3 mRNA levels, and 2) ^3^H-gemcitabine uptake within HEK293 cells.

In the clinic, using greater ultrasound pressures to increase transfection of tumor cells may not be a major issue since the goal is to eradicate the tumor and any cell death caused by the ultrasound would help treat the patient. Using focused ultrasound to transfect tumors would be ideal since it would decrease ultrasound energy deposition near the skin (causing low levels of toxicity) and increase ultrasound energy deposition within the tumor (causing higher levels of cell death and transfection). It may also be possible to increase transfection efficiency by complexing injected DNA with cationic polymers. Several studies have demonstrated that cell transfection can be increased synergistically if ultrasound and LSM as well as polyethylenimine are used for transfection [24], [25]. One potential issue with using polymers would be the lack of spatial control for transfection which is inherent with ultrasound and LSM.

Some of our mice died shortly after IV injection of LSM, presumably from gas embolisms. This issue has also been reported by other researchers [26] and has been attributed to spurious large bubbles. To our knowledge, this issue is only present in mice. LSM are clinically used as ultrasound contrast agents and are considered safe for humans. This issue led us to inject LSM inside tumors (instead of systemic administration by intravenous injection) preventing uniform transfection throughout the tumor and causing difficulty performing *in vivo* tumor growth inhibition assays since only a very small proportion of the tumor (cells near the needle tract) can become transfected. We demonstrated that injection of LSM and DNA inside tumors and subsequent ultrasound exposure can be used to transfect tissues/tumors. *In vivo* experiments of our proposed therapeutic strategy are still required. Future studies using targeted microbubbles or promising phase-change nanodroplets [27] may offer significant advantages compared to untargeted-LSM.

In the current study, cell viability with gemcitabine was assessed with HEK293 cells transfected with lipofectamine 2000 instead of ultrasound and LSM. We used the MTS assays for analyzing cell viability which analyzes cell metabolism for all cells within each well. Therefore, transfection of the minority of cells within wells transfected with ultrasound and LSM would likely not provide noticeable changes with the MTS assays. Lipofectamine 2000 provided transfection of ∼50% of HEK293 cells within each well and thus was a better transfection method to determine if hCNT3 cDNA transfection could reverse gemcitabine resistance in cells with little hENT activity using the MTS assay. Compared to HEK293 cells, AsPC-1 and MIA PaCa-2 cells had significantly lower transfection efficiency rates using lipofectamine (21% and 25%, respectively) and thus did not provide useful gemcitabine toxicity data using the MTS assay.

HEK293 cells expressed significant levels of hENT1 and hENT2 mRNA which were approximately 10- and 50-fold lower, respectively, than mRNA levels of the high expression “housekeeping gene” GAPDH. Due to the abundance of hENT1 and hENT2 mRNA transcript levels, untransfected HEK293 cells without dilazep incubation were relatively sensitive to gemcitabine with an EC_50_ value of 26±2 nM which is comparable to the EC_50_ values of other gemcitabine sensitive cell lines [28]. Inhibition of hENT1 and hENT2 with dilazep increased gemcitabine resistance by 13-fold. This is understandable since the only other nucleoside transporters in HEK293 capable of efficiently transporting gemcitabine were hCNT1 and hCNT3 which displayed 1,600- and 2,500-fold lower mRNA levels than that of hENT1, respectively (i.e., hCNT1/3 expression was very low in HEK293 cells). Transfection of hCNT3 in HEK293 cells incubated in dilazep (i.e., lacking hENT activity) was capable of sensitizing HEK293 cells to gemcitabine, suggesting that hCNT3 is capable reversing gemcitabine resistance in cells deficient of nucleoside transporter activity.

Although lipofectamine only transfected approximately half of the HEK293 cells in the wells, the gemcitabine EC_50_ values for HEK293 cells with functional hENTs (no dilazep) and HEK293 cells that had been transfected with hCNT3 and subsequently treated with dilazep to inhibit hENTs were very similar (EC_50_ values 26±2 and 35±10 µM, respectively, *P* = 0.49), suggesting that there may have been a bystander killing effect. In theory, transport deficient cells (e.g., in pancreatic cancers) transfected with hCNT3 would have high levels of intracellular gemcitabine and the apoptotic bodies from these cells should also have high levels of gemcitabine. Uptake of these apoptotic bodies by nearby nucleoside transport deficient cells may be a significant source of gemcitabine uptake for these cells. Intracellular gemcitabine may also have been transferred between HEK293 cells through gap junctions which are present in HEK293 [29], [30]. It is also possible that hCNT3 transfected cells may have released significant amounts of gemcitabine-containing exosomes which were then taken up by untransfected cells [31], [32]. Further experiments would be necessary to test these theories.

Transfection of hCNT3 cDNA in HEK293 cells incubated in dilazep increased gemcitabine uptake by >3400-fold compared to untransfected cells incubated in dilazep. However, under similar conditions, transfection of hCNT3 in AsPC-1 and MIA PaCa-2 cells increased gemcitabine uptake by approximately an order of magnitude less than that of HEK293 cells. This discrepancy may be explained if HEK293 cells shared cytoplasmic contents via gap junctions and/or exosomes. Untransfected HEK293 cells may still have large increases in gemcitabine uptake if nearby transfected cells expressed hCNT3, meaning our “transfection-corrected” gemcitabine uptake values for HEK293 cells may not be directly comparable to other cell lines which do not share cytoplasmic contents. Regardless, for all cell lines tested, hCNT3 transfection increased gemcitabine uptake 142- to 434-fold in cells lacking hENT activity compared to cells with functional hENTs.

**Figure 9:**
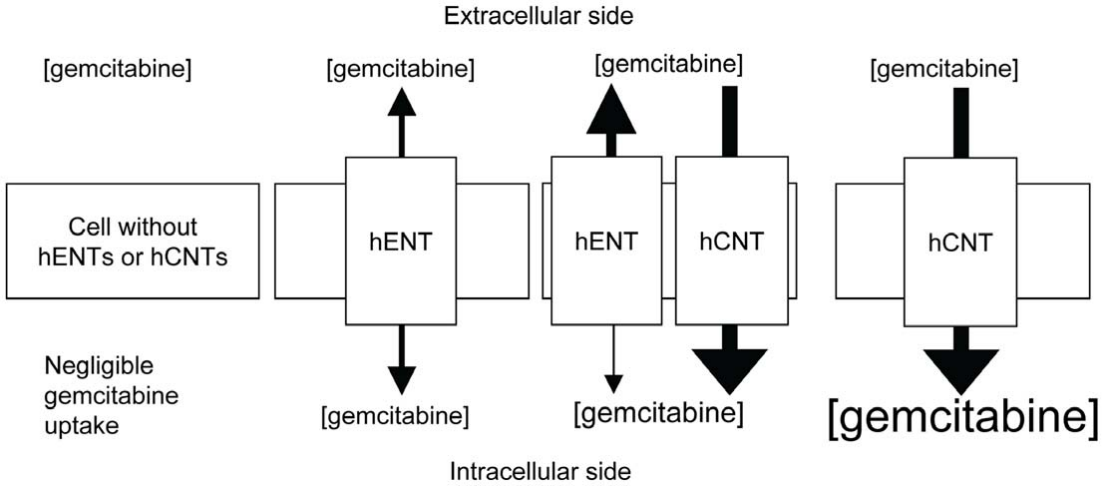
Model of gemcitabine uptake by nucleoside transporters. Gemcitabine is a hydrophilic drug that requires nucleoside transporters (hENT1/2 or hCNT1/3) for efficient uptake. hENTs can equilibrate gemcitabine levels across membranes but cannot actively accumulate the drug within cells. hCNT3 can use the Na^+^ (or H^+^ for hCNT3) cation transmembrane gradient to accumulate greater levels of gemcitabine within cells. For cells with significant levels of both hENTs and hCNTs, hCNTs actively transport gemcitabine within cells but hENTs will primarily efflux gemcitabine to equilibrate drug levels across membranes, causing reduced gemcitabine uptake compared to cells with only hCNTs. hENT1-negative cancer cells (which correlate with gemcitabine resistance) are presumed to have relatively low hENT activity such that transfection of these cells with a hCNT would be ideal for increasing gemcitabine uptake.

Transfection of hCNT3 in HEK293 cells with functional hENTs increased ^3^H-gemcitabine uptake by 8-fold. However, HEK293 cells transfected with hCNT3 cDNA and subsequently incubated with dilazep displayed 18-fold greater ^3^H-gemcitabine uptake than transfected cells with functional hENTs, suggesting that the presence of functional hENTs caused a *decrease* in ^3^H-gemcitabine uptake. This result was verified with ^3^H-gemcitabine uptake assays in *Xenopus* oocytes expressing hENT1/hCNT3. We proposed that the presence of functional plasma membrane hENTs (*i.e.*, hENT1 and/or hENT2), which mediate bidirectional transport, would equilibrate gemcitabine levels across plasma membranes, thereby primarily effluxing gemcitabine out of cells that have high hCNT3 expression. Figure 9 provides a model describing gemcitabine uptake in cells with different levels of NTs.

When comparing the various NTs as potential therapeutic genes for increasing the uptake of toxic nucleoside analog drugs, hCNT3 has multiple advantages over the other NTs. Being a hCNT, it can concentrate nucleoside analog drugs within cells to a much greater extent than the hENTs. Compared to hCNT1, hCNT3 co-transports an additional sodium ion (two in total) with each nucleoside, theoretically providing additional electrochemical gradient force to sustain nucleoside transport. This may partly explain why oocytes expressing hCNT3 accumulated 8.9-fold greater ^3^H-gemcitabine compared to oocytes expressing hCNT1 [5]. hCNT3 is also the only hCNT capable of using protons as cations for driving nucleoside transport. Tumors may have regions with relatively low pH, providing additional cations for nucleoside transport. Finally, hCNT3 is the only hCNT capable of transporting both purine and pyrimidine nucleoside analog drugs, allowing hCNT3 to transport many clinical drugs including gemcitabine, cladribine, clofarabine, and fludarabine.

Our results suggest that hCNT3 transfection will have the greatest effect increasing gemcitabine uptake and toxicity in cells without any functional NTs (*i.e.*, cancer cells resistant to gemcitabine therapy due to low NT activity). Most, if not all, normal tissues have detectable hENT1/2, especially hENT1 [33]. Therefore using hCNT3 as a therapeutic gene to increase gemcitabine uptake should have relatively little effect increasing gemcitabine uptake and toxicity on normal tissues but a much larger effect on gemcitabine resistant tumors with low NT activity which are currently a clinical problem.

## References

[1] Toschi L, Finocchiaro G, Bartolini S, Gioia V, Cappuzzo F (2005) Role of gemcitabine in cancer therapy. Future Oncol 1: 7–17. 16555971 10.1517/14796694.1.1.7

[2] Mackey JR, Mani RS, Selner M, Mowles D, Young JD, et al (1998) Functional nucleoside transporters are required for gemcitabine influx and manifestation of toxicity in cancer cell lines. Cancer Res 58: 4349–4357. 9766663

[3] Baldwin SA, Beal PR, Yao SY, King AE, Cass CE, et al (2004) The equilibrative nucleoside transporter family, SLC29. Pflugers Arch 447: 735–743. 12838422 10.1007/s00424-003-1103-2

[4] Gray JH, Owen RP, Giacomini KM (2004) The concentrative nucleoside transporter family, SLC28. Pflugers Arch 447: 728–734. 12856181 10.1007/s00424-003-1107-y

[5] Damaraju VL, Mowles D, Yao S, Ng A, Young JD, et al (2012) Role of human nucleoside transporters in the uptake and cytotoxicity of azacitidine and decitabine. Nucleosides Nucleotides Nucleic Acids 31: 236–255. 22356238 10.1080/15257770.2011.652330

[6] Farrell JJ, Elsaleh H, Garcia M, Lai R, Ammar A, et al (2009) Human equilibrative nucleoside transporter 1 levels predict response to gemcitabine in patients with pancreatic cancer. Gastroenterology 136: 187–195. 18992248 10.1053/j.gastro.2008.09.067

[7] Giovannetti E, Del Tacca M, Mey V, Funel N, Nannizzi S, et al (2006) Transcription analysis of human equilibrative nucleoside transporter-1 predicts survival in pancreas cancer patients treated with gemcitabine. Cancer Res 66: 3928–3935. 16585222 10.1158/0008-5472.CAN-05-4203

[8] Marechal R, Mackey JR, Lai R, Demetter P, Peeters M, et al (2009) Human equilibrative nucleoside transporter 1 and human concentrative nucleoside transporter 3 predict survival after adjuvant gemcitabine therapy in resected pancreatic adenocarcinoma. Clin Cancer Res 15: 2913–2919. 19318496 10.1158/1078-0432.CCR-08-2080

[9] Michalski CW, Erkan M, Sauliunaite D, Giese T, Stratmann R, et al (2008) Ex vivo chemosensitivity testing and gene expression profiling predict response towards adjuvant gemcitabine treatment in pancreatic cancer. Br J Cancer 99: 760–767. 18728667 10.1038/sj.bjc.6604528 PMC2528151

[10] Spratlin J, Sangha R, Glubrecht D, Dabbagh L, Young JD, et al (2004) The absence of human equilibrative nucleoside transporter 1 is associated with reduced survival in patients with gemcitabine-treated pancreas adenocarcinoma. Clin Cancer Res 10: 6956–6961. 15501974 10.1158/1078-0432.CCR-04-0224

[11] Perez-Torras S, Garcia-Manteiga J, Mercade E, Casado FJ, Carbo N, et al (2008) Adenoviral-mediated overexpression of human equilibrative nucleoside transporter 1 (hENT1) enhances gemcitabine response in human pancreatic cancer. Biochem Pharmacol 76: 322–329. 18589402 10.1016/j.bcp.2008.05.011

[12] Veltkamp SA, Pluim D, van Eijndhoven MA, Bolijn MJ, Ong FH, et al (2008) New insights into the pharmacology and cytotoxicity of gemcitabine and 2′,2′-difluorodeoxyuridine. Mol Cancer Ther 7: 2415–2425. 18723487 10.1158/1535-7163.MCT-08-0137

[13] Lang TT, Selner M, Young JD, Cass CE (2001) Acquisition of human concentrative nucleoside transporter 2 (hcnt2) activity by gene transfer confers sensitivity to fluoropyrimidine nucleosides in drug-resistant leukemia cells. Mol Pharmacol 60: 1143–1152. 11641443 10.1124/mol.60.5.1143

[14] Lang TT, Young JD, Cass CE (2004) Interactions of nucleoside analogs, caffeine, and nicotine with human concentrative nucleoside transporters 1 and 2 stably produced in a transport-defective human cell line. Mol Pharmacol 65: 925–933. 15044622 10.1124/mol.65.4.925

[15] Hernot S, Klibanov AL (2008) Microbubbles in ultrasound-triggered drug and gene delivery. Adv Drug Deliv Rev 60: 1153–1166. 18486268 10.1016/j.addr.2008.03.005 PMC2720159

[16] Chen S, Ding JH, Bekeredjian R, Yang BZ, Shohet RV, et al (2006) Efficient gene delivery to pancreatic islets with ultrasonic microbubble destruction technology. Proc Natl Acad Sci U S A 103: 8469–8474. 16709667 10.1073/pnas.0602921103 PMC1482516

[17] Luo YK, Zhao YZ, Lu CT, Tang J, Li XK (2008) Application of ultrasonic gas-filled liposomes in enhancing transfer for breast cancer-related antisense oligonucleotides: an experimental study. J Liposome Res 18: 341–351. 18985510 10.1080/03639040802509868

[18] Paproski RJ, Ng AM, Yao SY, Graham K, Young JD, et al (2008) The role of human nucleoside transporters in uptake of 3′-deoxy-3′-fluorothymidine. Mol Pharmacol 74: 1372–1380. 18669604 10.1124/mol.108.048900

[19] Livak KJ, Schmittgen TD (2001) Analysis of relative gene expression data using real-time quantitative PCR and the 2(-Delta Delta C(T)) Method. Methods 25: 402–408. 11846609 10.1006/meth.2001.1262

[20] Cass CE, Young JD, Baldwin SA (1998) Recent advances in the molecular biology of nucleoside transporters of mammalian cells. Biochem Cell Biol 76: 761–770. 10353709 10.1139/bcb-76-5-761

[21] Li YS, Davidson E, Reid CN, McHale AP (2009) Optimising ultrasound-mediated gene transfer (sonoporation) in vitro and prolonged expression of a transgene in vivo: potential applications for gene therapy of cancer. Cancer Lett 273: 62–69. 18829156 10.1016/j.canlet.2008.07.030

[22] Rahim AA, Taylor SL, Bush NL, ter Haar GR, Bamber JC, et al (2006) Spatial and acoustic pressure dependence of microbubble-mediated gene delivery targeted using focused ultrasound. J Gene Med 8: 1347–1357. 16981246 10.1002/jgm.962

[23] Tlaxca JL, Anderson CR, Klibanov AL, Lowrey B, Hossack JA, et al (2010) Analysis of in vitro transfection by sonoporation using cationic and neutral microbubbles. Ultrasound Med Biol 36: 1907–1918. 20800945 10.1016/j.ultrasmedbio.2010.05.014 PMC2996233

[24] Qiu Y, Luo Y, Zhang Y, Cui W, Zhang D, et al (2010) The correlation between acoustic cavitation and sonoporation involved in ultrasound-mediated DNA transfection with polyethylenimine (PEI) in vitro. J Control Release 145: 40–48. 20398711 10.1016/j.jconrel.2010.04.010

[25] Deshpande MC, Prausnitz MR (2007) Synergistic effect of ultrasound and PEI on DNA transfection in vitro. J Control Release 118: 126–135. 17258835 10.1016/j.jconrel.2006.12.010 PMC1941716

[26] Tsunoda S, Mazda O, Oda Y, Iida Y, Akabame S, et al (2005) Sonoporation using microbubble BR14 promotes pDNA/siRNA transduction to murine heart. Biochem Biophys Res Commun 336: 118–127. 16125678 10.1016/j.bbrc.2005.08.052

[27] Sheeran PS, Luois S, Dayton PA, Matsunaga TO (2011) Formulation and acoustic studies of a new phase-shift agent for diagnostic and therapeutic ultrasound. Langmuir 27: 10412–10420. 21744860 10.1021/la2013705 PMC3164903

[28] Paproski R, Young J, Cass C (2010) Predicting gemcitabine transport and toxicity in human pancreatic cancer cell lines with the positron emission tomography tracer 3′-deoxy-3′-fluorothymidine. Biochem Pharmacol 79: 587–595. 19788890 10.1016/j.bcp.2009.09.025

[29] Butterweck A, Gergs U, Elfgang C, Willecke K, Traub O (1994) Immunochemical characterization of the gap junction protein connexin45 in mouse kidney and transfected human HeLa cells. J Membr Biol 141: 247–256. 7807524 10.1007/BF00235134

[30] Del Re AM, Woodward JJ (2005) Inhibition of gap junction currents by the abused solvent toluene. Drug Alcohol Depend 78: 221–224. 15845326 10.1016/j.drugalcdep.2004.10.005

[31] Pant S, Hilton H, Burczynski ME (2012) The multifaceted exosome: biogenesis, role in normal and aberrant cellular function, and frontiers for pharmacological and biomarker opportunities. Biochem Pharmacol 83: 1484–1494. 22230477 10.1016/j.bcp.2011.12.037 PMC7110994

[32] Sokolova V, Ludwig AK, Hornung S, Rotan O, Horn PA, et al (2011) Characterisation of exosomes derived from human cells by nanoparticle tracking analysis and scanning electron microscopy. Colloids Surf B Biointerfaces 87: 146–150. 21640565 10.1016/j.colsurfb.2011.05.013

[33] Pennycooke M, Chaudary N, Shuralyova I, Zhang Y, Coe IR (2001) Differential expression of human nucleoside transporters in normal and tumor tissue. Biochem Biophys Res Commun 280: 951–959. 11162617 10.1006/bbrc.2000.4205

